# Disordered gaming, loneliness, and family harmony in gamers before and during the COVID-19 pandemic

**DOI:** 10.1016/j.abrep.2022.100426

**Published:** 2022-04-12

**Authors:** Dmitri Rozgonjuk, Halley M. Pontes, Bruno Schivinski, Christian Montag

**Affiliations:** a Department of Molecular Psychology, Institute of Psychology and Education, Ulm University, Ulm, Germany; b Institute of Mathematics and Statistics, University of Tartu, Tartu, Estonia; c Department of Organizational Psychology, Birkbeck, University of London, London, United Kingdom; d School of Media and Communication, RMIT University, Melbourne, VIC, Australia

**Keywords:** Gaming disorder, Internet gaming disorder, Loneliness, Family harmony, COVID-19, Pandemic

## Abstract

The aim of this study was to investigate if and how disordered gaming, loneliness, and family relations have changed throughout the COVID-19 pandemic (from 2019 to 2021), and whether there were any changes in the association between these variables across three samples of gamers (for each respective year). Samples from 2019, 2020, and 2021 were matched by using propensity score matching across socio-demographic characteristics. The total effective sample comprised 897 gamers (N = 299 per year). These samples were compared in terms of disordered gaming – separately as Gaming Disorder (GD; WHO framework) and Internet Gaming Disorder (IGD; APA framework), loneliness, and family harmony scores with analyses of covariance (ANCOVAs), with age and gender as covariates. Steiger tests were used for correlation differences testing. ANCOVAs showed that while GD and IGD scores have increased significantly during the pandemic years, loneliness and family harmony did not change significantly. Furthermore, the correlation differences tests indicated that the correlations between both IGD and GD with loneliness as well as poorer family harmony have increased during the pandemic years. This study provides empirical evidence that the well-being of gamers might have been negatively affected during the COVID-19 pandemic. While loneliness and family harmony did not increase, the stronger correlations between Gaming Disorder and other variables might suggest that gaming may have been used to cope with loneliness and poorer family harmony.

## Highlights

• (Internet) Gaming Disorder scores increased significantly during the pandemic years.
• Loneliness and family harmony did not change significantly.
• Correlations between (I)GD and loneliness/poorer family harmony increased.
• The COVID-19 pandemic might have negatively affected the well-being of gamers.

## 1 Introduction

The COVID-19 pandemic had many highly influential effects on societies across the world. Several measures were adopted by different countries worldwide to cope with the pandemic and to prevent further strain on the health of individuals as well as medical systems. Arguably, one of the most important societal restrictions was enforcing physical distancing limiting physical contact/proximity between people to curb infection rates (MacIntyre & Wang, 2020). These restrictions also introduced changes in education and professional activities by facilitating (if not mandating) studying and working from home. Similarly, leisure was also impacted, as social activities with multiple people in close physical proximity were also discouraged and/or made illegal.

The restrictions introduced due to the COVID-19 pandemic conditions led people to find compatible leisure activities, such as electronic gaming as it can be engaged without other people physically present. As a result, several campaigns were promoted during the pandemic to encourage people to play video games whilst staying at home safe (e.g., Play at Home, #PlayApartTogether). Furthermore, several studies (e.g., described in King et al., 2020, Vuorre et al., 2021) have demonstrated that gaming has escalated significantly during the pandemic, with recent research suggesting that increased gaming may be due to pandemic-related stress (Balhara et al., 2020, Shrestha et al., 2020). Interestingly, Teng et al. (2021) conducted a longitudinal study of 1,778 children and adolescents in China, examining the relationship between psychological health and disordered gaming before (i.e., 2019) and during the pandemic (i.e., 2020). They concluded that while playing video games increased in both children and adolescents during the pandemic, only adolescents reported greater levels of disordered gaming symptoms during the pandemic. However, it should be noted that gaming, especially during the pandemic, might not necessarily be detrimental. Empirical evidence shows that gaming activities have risen during the pandemic – this applies to gaming in general (Vuorre et al., 2021), but also to disordered gaming (King et al., 2020). Thus, it can be argued that the increase of gaming was further fueled by highly engaging gaming features and game releases driven by the rising market during the pandemic (López-Cabarcos et al., 2020). Furthermore, Barr & Copeland-Stewart (2022) also outlined the potential beneficial effects of gaming, as this activity provides cognitive stimulation and opportunities to socialise with others. For instance, associations with gaming's stress and anxiety reducing functions were also found (Barr & Copeland-Stewart, 2022), suggesting that gaming might be used to alleviate these negative affective states (the so-called “self-medication” hypothesis).

Physical contact restrictions reduce social need satisfaction which could result in experiencing loneliness (Heinrich & Gullone, 2006); in fact, better social need satisfaction is also linked to less problematic engagement in Internet-related activities (Rozgonjuk, Davis, & Montag, 2021). Loneliness is a significant psychological construct, as it has high clinical relevance in predicting psychopathology (Heinrich & Gullone, 2006), such as depression, sleep disorders (Mushtaq et al., 2014) as well as suicidal ideation (McClelland et al., 2020) – disorders that showed elevations during the pandemic (Brailovskaia et al., 2021, Brailovskaia et al., 2021, Gelezelyte et al., 2021). Studies have found that, in comparison to pre-pandemic years, individuals have reported elevated levels of loneliness during the COVID-19 pandemic (Groarke et al., 2020, Killgore et al., 2020, Li and Wang, 2020). Recent findings have shown that experiencing loneliness during the pandemic is associated with younger age, female gender, lower socio-economic status, living alone, lower perceived social support, and past or current COVID-19 symptoms (Bu et al., 2020, Groarke et al., 2020). This is important, as those at greater risk for loneliness were even at higher risk to experience loneliness during the pandemic (Bu et al., 2020).

Emotional support from family is associated with experiencing less loneliness (Taniguchi & Kaufman, 2021). Relatedly, adequate family relationships are necessary for building trust, increasing social cohesion, and subjective well-being (Gayatri & Irawaty, 2021). Adequate family relationships also help to protect against pandemic-related emotional distress (Dong et al., 2020). Several recent studies indicate that the pandemic is associated with decreased well-being in family relationships. For instance, parents have reported more irritability, less positive expressiveness, higher levels of alcohol consumption (Westrupp et al., 2021) as well as general decline in relationship quality, intimacy, and mental health (Goldberg et al., 2021). It may, therefore, not come as a surprise that higher domestic violence rates have been observed during the pandemic (Bradbury-Jones and Isham, 2020, Sediri et al., 2020, Zhu et al., 2021), and that greater incidence of loneliness has also been reported (Groarke et al., 2020, Killgore et al., 2020, Li and Wang, 2020).

Social isolation (e.g., feeling lonely and/or detached from family) may motivate people to engage more in gaming to cope with isolation. Importantly, greater time spent on gaming has been shown to be associated with gaming for social reasons, but also to relieve stress in daily-life (de Hesselle et al., 2021). This is in line with findings from pandemic-studies reported above, as gaming in general has increased, with some studies suggesting the pandemic-induced stress as the root cause (Balhara et al., 2020, Shrestha et al., 2020). In some cases, however, “self-medication” via gaming may drive non-adaptive behavioral patterns of excessive gaming that could lead to problems in daily-life (Brand et al., 2019). In other words, excessive time spent on gaming leads to disordered gaming in some gamers (Pontes et al., 2022).

Disordered gaming reflects both Gaming Disorder (GD) and Internet Gaming Disorder (IGD). GD represents an officially recognized mental health disorder within the World Health Organization framework (WHO; World Health Organization, 2018), while IGD has been put forward by the American Psychiatric Association framework (APA; American Psychiatric Association, 2013) as a tenative diagnosis in 2013. There are several symptoms underpinning disordered gaming that are related to excessive gaming (e.g., loss of control over gaming, prioritizing gaming over other relevant life activities, etc.) leading to daily-life impairment and decreased health (Männikkö et al., 2020, Moore et al., 2022, Pontes, 2017). Disordered gaming has been linked to loneliness (Montag et al., 2021, Zeliha, 2019), depression (King et al., 2019, Montag et al., 2021), health anxiety (Elhai et al., 2021), eating disorders (Micallef et al., 2021), as well as other digital technology based problematic behaviors (Rozgonjuk et al., 2021). Importantly, at a family level, disordered gaming has been linked to poorer family relationships (Şahin et al., 2019) and less harmony in the family (Ekşi et al., 2020). Relatedly, a recent study demonstrated that family support decreases loneliness which, in turn, reduces disordered gaming (Şahin et al., 2019).

Although most of the findings thus far discussed have primarily explored the associations within the general public, little is known about how the COVID-19 pandemic is associated with gamers' psychological health. Hence, the aim of the present study is to fill that gap. Specifically, this study examines the interplay between disordered gaming, loneliness, and family harmony across three years: pre-pandemic (2019) and during the initial two years of the COVID-19 pandemic (2020, 2021). In order to contribute to the emerging comparative research on disordered gaming, the present study adopts the WHO and APA frameworks as they may lead to different disordered gaming prevalence rates in study populations (Montag et al., 2019, Montag et al., 2021, Pontes et al., 2022). Based on the literature discussed above, the following two hypotheses will be tested in light of the WHO and APA frameworks, hence:

H1: *Disordered gaming, loneliness, and dysfunctional family harmony will increase during the pandemic years (2020 and 2021) compared to the pre-pandemic year (2019).*

H2: *The relationship between disordered gaming and loneliness and family harmony will be stronger during the pandemic years (2020 and 2021) compared to the pre-pandemic year (2019).*

## 2 Methods

### 2.1 Sample and procedure

The data were retrieved from a larger international online survey project among others promoted via the ‘Smart Gaming’ campaign (see https://about.eslgaming.com/portfolio/smart-gaming), which facilitated this independent piece of research where the main focus was on promoting responsible gaming. The study has been active since 2019, and the data were also collected in 2020 and 2021. Although the study language was English, there were no restrictions with regards to a participant’s country of origin as all participants were proficient in English. The survey platform was advertised via multiple channels (e.g., webpages, specialized forums, online news channels, etc.). Prior to study participation, all potential participants were required to pass the eligibility criteria (see below) and to provide electronic informed consent. Participation was anonymous and the main incentive for participation in the study was the possibility to receive feedback on one’s gaming behaviors in comparison to aggregated scores of other participants. Receiving accurate personalized feedback was also used to gain truthful responses. All study procedures were carried out in accordance with contemporary ethical standards, and the study project was approved by the research team’s University Ethics Committee (PONTES 2018/95, Nottingham Trent University).

The sample used for the present study included respondents who provided a valid informed consent; were in the age range of 12–80 years; reported sufficient proficiency in English; had played video games over the past year; passed the attention check item (responded negatively to playing a fictional computer game); reported not being professional gamers; spent <=119 h per week in gaming; reported spending <=48 h of gaming on weekends (Saturday and Sunday); had responded to the variables of interest. As a result, this amounted to a sample size of 47,503 gamers. However, to be able to draw meaningful comparisons across different years, we only included the responses with comparable age range (12–56 years) and represented countries (at least five respondents from a given country in 2020 and 2021). This resulted in a sample size of 37,394 gamers (N_2019_ = 36,078, N_2020_ = 1017, N_2021_ = 299). Since the sample was heterogeneous with regards to country, and there were high imbalances in sample size, we used propensity score matching (see *Analysis* section for details) to match the samples grouped by response year to balance (and match) the samples. Samples from 2019 and 2020 were matched with the 2021 sample (N = 299) by age, gender, country, education level (no high school degree, high school degree, university/college degree), employment status (employed vs unemployed), and relationship status (in or not in a relationship). The seed that helps to create the replicate of the random generation was set to 999 in all functions implementing random generation. As a result, the effective sample size was N = 897. The breakdown of the sample socio-demographics by each year is shown in Table 1. Please note that data from 2019 has been analyzed regarding different research questions such as links between personality and Gaming Disorder or time spent on gaming and Gaming Disorder in other recent works (Montag et al., 2021, Pontes et al., 2022). Moreover, further variables have been assessed in the survey giving insights into topics such as professional gaming, gaming motives, etc., which are not part of the present work, but will be investigated in the near future.

**Table 1:** Sample socio-demographics grouped by each year.

		**Year**	
**Variable**	**2019 (N = 299)**	**2020 (N = 299)**	**2021 (N = 299)**
**Age**			
Mean (SD)	24.45 (7.31)	24.37 (7.51)	25.23 (8.37)

**Gender**			
Male	257 (85.95%)	251 (83.95%)	246 (82.27%)
Female	42 (14.05%)	48 (16.05%)	53 (17.73%)

**Employment status**			
Employed	140 (46.82%)	134 (44.82%)	139 (46.49%)

**Relationship status**			
In a relationship	115 (38.46%)	109 (36.45%)	114 (38.13%)

**Education level**			
< High school	43 (14.38%)	46 (15.38%)	37 (12.37%)
High school diploma	150 (50.17%)	147 (49.16%)	157 (52.51%)
College/university degree	106 (35.45%)	106 (35.45%)	105 (35.12%)

**Top five countries (by total N)**			
France	33 (11.04%)	32 (10.070%)	31 (10.37%)
Italy	30 (10.03%)	33 (11.04%)	30 (10.03%)
USA	24 (8.03%)	29 (9.70%)	26 (8.70%)
Spain	22 (7.36%)	32 (10.70%)	21 (7.02%)
Poland	23 (7.69%)	22 (7.36%)	25 (8.36%)

### 2.2 Measures

#### 2.2.1 Disordered gaming

Internet Gaming Disorder (IGD) was assessed with the nine-item Internet Gaming Disorder Scale – Short-Form (IGDS9–SF; Pontes & Griffiths, 2015). Gaming Disorder (GD) was assessed with the four-item Gaming Disorder Test (GDT; Pontes et al., 2019). In both scales, the response options range from 1 = “never” to 5 = “very often”. Total scores can be obtained by summation and higher scale scores reflect higher symptom severity of disordered gaming. Cronbach's alphas for the effective sample were α = 0.87 (IGDS9-SF) and α = 0.81 (GDT).

#### 2.2.2 Dysfunctional family harmony

Family harmony was assessed with the five-item, unidimensional Family Harmony Scale (FHS-5) developed by Kavikondala et al. (2016). The FHS-5 scale score reflects the extent of family functioning, interaction peacefulness, and harmony of one's family. The responses for items range from 1 = “strongly agree” to 5 = “strongly disagree”. The scale score is summed to form an index of dysfunctional family harmony where higher scores reflect more dysfunctional family harmony. The internal consistency of the scale for the effective sample in the current study was Cronbach's α = 0.90.

#### 2.2.3 Loneliness

We used the short, three-item UCLA Loneliness Scale (ULS-3; Hughes et al., 2004) to assess the frequency of loneliness experienced by study participants. For each item, the responses range from 1 = “never” to 4 = “often”. Summed score is used to reflect the extent of experienced loneliness, with higher scores indicating more loneliness experienced. Cronbach's α for the effective sample was 0.83.

### 2.3 Analysis

Data analysis was conducted in R v4.1.3. (R Core Team, 2022); all used packages are reported in Supplementary Materials. First, the samples across years were matched by socio-demographic characteristics (described in *Sample and Procedure*); greedy nearest neighbor matching with logistic regression was used to estimate propensity scores. Analyses of covariance were used for testing differences in outcomes (i.e., GD, IGD, family harmony, and loneliness scores) across years; age and gender were included as covariates. Holm's post hoc corrections were used for pairwise comparisons. Spearman partial correlation analysis (*p*-values corrected with Holm's method) was used to investigate the associations between GD, IGD, family harmony, and loneliness for each year with age treated as covariate. This analysis was followed by (partial) correlation differences significance testing (Steiger tests), where given correlations were contrasted across two given years/samples.

## 3 Results

### 3.1 Descriptive statistics and differences across years

**Fig. 1:**
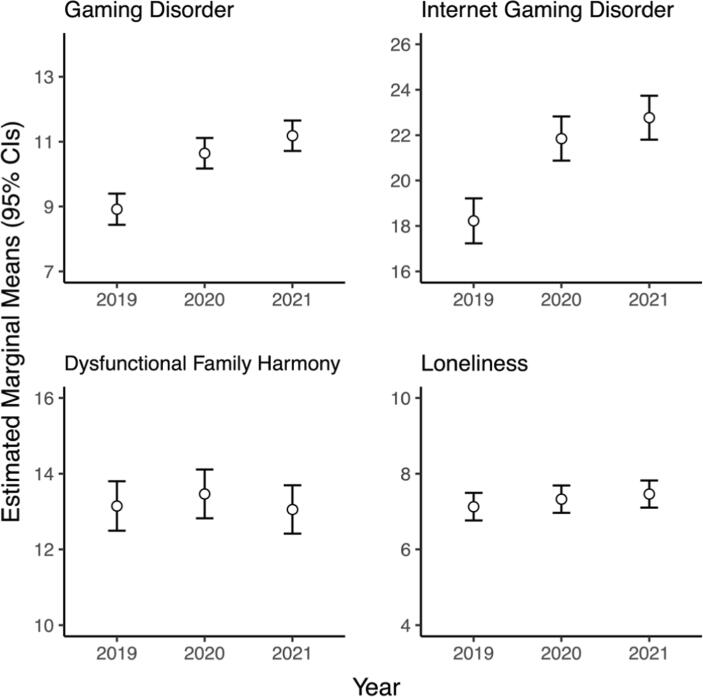
Estimated marginal means (with 95% CIs) for variables across years (controlled for age and gender).

The descriptive statistics are presented in Table 2. Results of analyses of covariance for each year where each key variable is the dependent variable is in Table 3, Holm's post-hoc comparisons are in Table 4. Fig. 1 provides a graphical depiction of estimated marginal means (with 95% CIs) for variables. Comparing changes in scores across years could be helpful in identifying whether there have been changes in GD and IGD symptomatology, as well as dysfunctional family harmony and experiencing loneliness.

**Table 2:** Descriptive statistics for key variables.

	**Year**					
	**2019**		**2020**		**2021**	
**Variable**	**M**	**SD**	**M**	**SD**	**M**	**SD**
GD	8.96	3.30	10.69	3.81	11.18	3.95
IGD	18.29	6.54	21.92	8.01	22.80	8.09
DFH	12.76	5.24	13.10	4.96	12.67	4.90
Loneliness	6.89	2.80	7.11	2.82	7.24	2.83

Notes. M = Mean; SD = Standard Deviation; GD = Gaming Disorder; IGD = Internet Gaming Disorder; DFH = Dysfunctional Family Harmony.

**Table 3:** Results of analyses of covariance.

	**Outcome: Gaming Disorder (WHO Framework)**					
**Variable**	**SS**	**df**	**MS**	**F**	**p**	**η^2^p**
Year	837.388	2	418.694	30.986	**< 0.001**	0.065
Age	152.079	1	152.079	11.255	**< 0.001**	0.012
Gender	0.972	1	0.972	0.072	0.789	< 0.001
Residuals	12053.169	892	13.513			

	**Outcome: Internet Gaming Disorder (APA Framework)**					
**Variable**	**SS**	**df**	**MS**	**F**	**p**	**η^2^p**
Year	3445.648	2	1722.824	30.000	**< 0.001**	0.063
Age	131.046	1	131.046	2.282	0.131	0.003
Gender	3.156	1	3.156	0.055	0.815	< 0.001
Residuals	51228.114	892	57.431			

	**Outcome: Loneliness**					
**Variable**	**SS**	**df**	**MS**	**F**	**p**	**η^2^p**
Year	16.922	2	8.461	1.074	0.342	0.002
Age	11.898	1	11.898	1.510	0.219	0.002
Gender	53.285	1	53.285	6.762	**0.009**	0.008
Residuals	7029.120	892	7.880			

	**Outcome: Dysfunctional Family Harmony**					
**Variable**	**SS**	**df**	**MS**	**F**	**p**	**η^2^p**
Year	27.769	2	13.884	0.552	0.576	0.001
Age	92.869	1	92.869	3.689	0.055	0.004
Gender	149.283	1	149.283	5.930	**0.015**	0.007
Residuals	22454.191	892	25.173			

*Notes*. SS = sum of squares; MS = mean square; η^2^p = partial eta-squared.

**Table 4:** Outcome variable comparisons across years (controlled for age and gender).

	**Outcome: Gaming Disorder**				
**Comparison**	**MD**	**SE**	**t**	**p**	**Cohen's d**
2019–2020	−1.727	0.301	−5.742	**< 0.001**	−0.470
2019–2021	−2.269	0.301	−7.534	**< 0.001**	−0.617
2020–2021	−0.542	0.301	−1.802	0.072	−0.148

	**Outcome: Internet Gaming Disorder**				
**Comparison**	**MD**	**SE**	**t**	**p**	**Cohen's d**
2019–2020	−3.625	0.620	−5.847	**< 0.001**	−0.478
2019–2021	−4.546	0.621	−7.323	**< 0.001**	−0.600
2020–2021	−0.921	0.621	−1.485	0.138	−0.122

	**Outcome: Loneliness**				
**Comparison**	**MD**	**SE**	**t**	**p**	**Cohen's d**
2019–2020	−0.199	0.230	−0.869	0.771	−0.071
2019–2021	−0.335	0.230	−1.456	0.437	−0.119
2020–2021	−0.135	0.230	−0.589	0.771	−0.048

	**Outcome: Dysfunctional Family Harmony**				
**Comparison**	**MD**	**SE**	**t**	**p**	**Cohen's d**
2019–2020	−0.319	0.410	−0.777	0.952	−0.064
2019–2021	0.092	0.411	0.224	0.952	0.018
2020–2021	0.411	0.411	1.000	0.952	0.082

*Notes*. In all comparisons, df = 892. MD = mean difference. P-values adjusted with Holm's method. P-values for statistically significant differences are highlighted in **bold** font.

Table 3 shows that statistically significant differences between years can be observed in GD and IGD scores, whereas there are no clear associations between survey year/sample and loneliness and dysfunctional family harmony. Furthermore, age is a significant covariate in the model with GD as the outcome, while gender is a significant covariate for family harmony and experiencing loneliness.

Holm's post hoc tests (Table 4) show that within GD and IGD, the differences between pre-pandemic (2019) and pandemic (2020, 2021) years stand out. Specifically, GD and IGD scores during 2019 were lower than in 2020 and 2021, with medium effect sizes (Cohen, 1988).

### 3.2 Correlation analysis and correlation differences

The results of the Spearman partial correlation analysis (age treated as covariate) and correlation differences tests are presented in Table 5. These results help informing (1) if and how strongly given constructs are associated with each other (controlled for age) and (2) if the association strengths have changed between pre- (2019) and during the two initial pandemic years (2020, 2021).

**Table 5:** Spearman correlation coefficients and correlation differences test results.

	**Correlations**			**Comparisons**					
	**2019**	**2020**	**2021**	**2019 – 2020**		**2019 – 2021**		**2020 – 2021**	
**Variables**	**rho**	**rho**	**rho**	**z**	**p**	**z**	**p**	**z**	**p**
GD & IGD	0.751***	0.800***	0.802***	1.501	0.133	1.569	0.117	0.068	0.946
GD & DFH	0.121*	0.122*	0.305***	0.012	0.990	2.353	**0.01**9	2.341	**0.01**9
GD & LON	0.207**	0.346***	0.393***	1.835	0.066	2.498	**0.01**3	0.662	0.508
IGD & DFH	0.176**	0.143*	0.307***	0.412	0.680	1.696	0.090	2.108	**0.0**35
IGD & LON	0.293***	0.427***	0.463***	1.878	0.060	2.424	**0.01**5	0.546	0.585
DFH & LON	0.253***	0.224***	0.311***	0.374	0.708	0.767	0.443	1.141	0.254

*Notes*. GD = Gaming Disorder; IGD = Internet Gaming Disorder; DFH = Dysfunctional Family Harmony; LON = Loneliness. P-values for correlations corrected with Holm's method for each year. *** p <.001, ** p <.01, * p <.01. P-values for statistically significant correlation differences are highlighted in **bold** font.

As anticipated, GD and IGD scores yield a high positive correlation with each other (Montag et al., 2019), and the correlation change across the three years (ranging from r = 0.751 to r = 0.802) was not statistically significant. The correlation between loneliness and dysfunctional family harmony did not change over the three-year period and is positive and yields small-to-medium effect sizes (r = 0.224 to r = 0.311).

Table 5 shows that the correlation between loneliness and both GD and IGD has increased from 2019 to 2021, from r = 0.207 (GD) and r = 0.293 (IGD) in 2019 to r = 0.393 (GD) and r = 0.463 (IGD), respectively. Furthermore, it could be observed that the correlation between dysfunctional family harmony and GD and IGD has increased by 2021; for GD, the correlation increased from r = 0.121 (2019) to r = 0.305 (2021), and for IGD, the change was from r = 0.143 (2020) to r = 0.307 (2021). It should be noted that the correlation between dysfunctional family harmony and GD was roughly the same in 2019 and 2020, and hence the increase between dysfunctional family harmony-GD correlation could also be observed between 2019 and 2021 years. Interestingly, none of the correlations were significantly different between 2019 and 2020.

## 4 Discussion

The main goal of the current study was to investigate the interplay between disordered gaming (IGD and GD), loneliness, and family harmony in 2019 to 2021 (before and during the initial two years of the COVID-19 pandemic) among gamers. To reach this goal, we analyzed the responses of the respective years' samples in terms of changes in average scale scores as well as correlations.

Several studies have demonstrated that during the pandemic (in comparison to pre-pandemic years), there were significant increases in levels of gaming (Balhara et al., 2020, Shrestha et al., 2020, Vuorre et al., 2021), loneliness (Groarke et al., 2020, Killgore et al., 2020, Li and Wang, 2020), and dysfunctional family relations (Bradbury-Jones and Isham, 2020, Goldberg et al., 2021, Westrupp et al., 2021, Zhu et al., 2021). Based on the existing literature, we expected to observe similar patterns in a sample of gamers. In other words, we hypothesized that the scores of disordered gaming (IGD and GD), loneliness, and dysfunctional family harmony would have increased due to the COVID-19 pandemic restrictions and inflicted social limitations in comparison to pre-pandemic (i.e., 2019). The results showed that while disordered gaming scores increased, there were no statistically significant differences in experiencing loneliness and poorer family harmony across the different years investigated (although loneliness levels rose at a descriptive level slightly over the investigated years).

Therefore, these results are partially supporting the first hypothesis (H1). It is, however, surprising that levels of loneliness and family harmony did not change before and during the pandemic (at least on a statistically significant level). Based on the existing literature, increased loneliness and more dysfunctional family relations would have been expected. Recently, disordered gaming has been associated with an average of about 34 h of gaming per week (when assessed with the APA diagnostic framework) and an average of about 40 h of gaming per week (when assessed with the WHO framework) (Pontes et al., 2022). However, in light of research reporting increased time spent on gaming during the pandemic (Vuorre et al., 2021), it could be argued that the COVID-19 global pandemic has created a particular environment that may lead to problematic behavior and subsequently to possible well-being problems, because restrictions may induce gaming. Coping with pandemic-induced stress could lead to relying on gaming as a stress-relief (also evidenced in Barr & Copeland-Stewart, 2022); which in turn could lead to subsequent disruption in daily life (e.g., arguments with others due to gaming, prioritizing gaming over other important daily activities, such as school or job). Yet, it does not seem that gamers were significantly lonelier or had their family relations deteriorated during this time. That could be explained by assuming that although the COVID-19 pandemic led to social restrictions, on average, individuals with close social relations were still able to live together as families. Whereas, emerging research revealed that risk factors for increasing loneliness during the pandemic were similar as in prepandemic cases (e.g. individuals living alone; Bu et al., 2020).

In terms of the second hypothesis (H2), it was anticipated that disordered gaming (GD and IGD) would be more strongly associated with loneliness and dysfunctional family relations during the pandemic years. The results showed that while the correlations did not differ from each other between 2019 and 2020, there were some significant differences between 2019 and 2021. Namely, the correlation between disordered gaming (both IGD and GD) and dysfunctional family harmony increased from 2020 to 2021 from small to medium effect sized association. These results could indicate to the possibility that even though the levels of dysfunctional family harmony did not increase, it may have contributed to (developing) disordered gaming more so than before. This finding is consistent with the notion that gaming during the pandemic may constitute a coping mechanism to relieve stressful factors (e.g., loneliness and poor family relationships). However, whether loneliness and poor family relations contributed to higher disordered gaming needs to be further studied.

The findings also show that there may be some differences in results based on whether the WHO's or APA's disordered gaming diagnostic framework is used. For example, while the association size between GD and dysfunctional family harmony increased from 2019 to 2021, a similar increase was not statistically significant between IGD and dysfunctional family harmony for the same period. This could partially be explained by a statistical artefact, whereas IGD and dysfunctional family harmony have a stronger association in 2019 (r = 0.176, while it was r = 0.121 for GD), since one can clearly notice that in 2021, the correlation size was increased for both IGD (r = 0.307) and GD (r = 0.305). Nevertheless, these differences could hint to different measures picking up different nuances in disordered gaming (Mõttus et al., 2017). Indeed differences have been shown in disordered gaming-outcomes studies, but the overall correlations appear to be of similar magnitude (Montag, Kannen, et al., 2021).

The current study provides several contributions to the field. First, it demonstrates that some sub-populations (in this case, gamers) may not necessarily experience the same dynamics in pandemic-related psychological strains as the general population. Second, the study provides evidence that disordered gaming (among gamers) has increased over the pandemic compared to the pre-pandemic year. Third, the results also show that loneliness and dysfunctional family relations might not have surged among gamers; however, these factors played a greater role in disordered gaming during the pandemic than before.

The limitations of the current study include using self-report questionnaires, cross-sectional study design, and using an online sample. While the study participants assessed the severity of daily-life impairment due to gaming, it would have been interesting to include objective measures regarding time spent gaming. Furthermore, we used independent samples of gamers to compare the interplay between gaming, loneliness, and family harmony; using a repeated-measures study design with the same participants could provide insights into intra-individual associations. Since the study used cross-sectional samples in all years, causal inferences of the results are limited and should be made with careful consideration. As previously described in this study (see the Introduction section), in 2020, highly immersive video games were released, which could also be a factor influencing our findings, aside from the pandemic. It is also noteworthy that most of the respondents in the present study were male, introducing potential gender bias. Although women comprise approximately half of the gaming population, there may be obstacles, such as stereotypes and role expectancies, for women playing video games (Lopez-Fernandez et al., 2019). Even though this is out of the scope of the current study, one could hypothesize that participation in the current survey might have been impacted by these biases. We also mention that some variables, such as family harmony, may be prone to age-effects, and this cannot be completely ruled out. Finally, our study relied on an online sample which may introduce self-selection bias; however, this is a potential limitation for likely most online surveys (Schivinski et al., 2018).

## Conflict of Interest

The authors report no conflict of interest.

However, for reasons of transparency Dr. Montag mentions that he has received (to Ulm University and earlier University of Bonn) grants from agencies such as the German Research Foundation (DFG). Dr. Montag has performed grant reviews for several agencies; has edited journal sections and articles; has given academic lectures in clinical or scientific venues or companies; and has generated books or book chapters for publishers of mental health texts. For some of these activities he received royalties, but never from gaming or social media companies. Dr. Montag mentions that he is part of a discussion circle (Digitalität und Verantwortung: https://about.fb.com/de/news/h/gespraechskreis-digitalitaet-und-verantwortung/) debating ethical questions linked to social media, digitalization and society/democracy at Facebook. In this context, he receives no salary for his activities. Finally, he mentions that he currently functions as independent scientist on the scientific advisory board of the Nymphenburg group (Munich, Germany). This activity is financially compensated. Moreover, he is on the scientific advisory board of Applied Cognition (Redwood City, CA, USA), an activity which is also compensated.

## CRediT authorship contribution statement

**Dmitri Rozgonjuk:** Conceptualization, Methodology, Formal analysis, Investigation, Writing – original draft. **Halley M. Pontes:** Project administration, Investigation, Writing – review & editing. **Bruno Schivinski:** Project administration, Writing – review & editing. **Christian Montag:** Conceptualization, Investigation, Project administration, Writing – review & editing.
