# Supplementary material for: Disordered gaming, loneliness, and family harmony in gamers before and during the COVID-19 pandemic
Source: Addict Behav Rep. 2022 Apr 12;15:100426. doi: 10.1016/j.abrep.2022.100426 (PMC9001174; doi:10.1016/j.abrep.2022.100426)
Supplement: Supplementary Data 1 [file mmc1.pdf]

Supplementary Materials

Statistical packages used for analyses

The following statistical packages were used: *tidyverse* v1.3.1 (Wickham et al., 2019) for general data processing; *psych* v2.2.3 (Revelle, 2021) for computing internal consistency statistics and correlation differences testing (Steiger Test); *jmv* v2.0 (Selker et al., 2020) for analyses of covariances, Holm's post-hoc comparisons (including effect size measures); *RcmdrMisc* v2.7.2 (Fox, 2022) for Spearman (partial) correlation analysis (p-values adjusted with Holm's method for each year); MatchIt v4.3.4 (Ho et al., 2011) for propensity score matching; *ggplot2* v3.3.5 (Wickham, 2016) and *patchwork* v1.1.1 (Pedersen, 2020) were used for plotting.

References

Fox, J. (2022). *RcmdrMisc: R Commander Miscellaneous Functions* (2.7-2) [Computer software]. https://CRAN.R-project.org/package=RcmdrMisc

Ho, D. E., Imai, K., King, G., & Stuart, E. A. (2011). MatchIt: Nonparametric Preprocessing for Parametric Causal Inference. *Journal of Statistical Software*, *42*(8). https://doi.org/10.18637/jss.v042.i08

Pedersen, T. L. (2020). *patchwork: The Composer of Plots* (1.1.1) [Computer software]. https://CRAN.R-project.org/package=patchwork

Revelle, W. (2021). *psych: Procedures for personality and psychological research.* (2.2.3) [Computer software]. https://CRAN.R-project.org/package=psych

Selker, R., Love, J., & Dropmann, D. (2020). *jmv: The "jamovi" Analyses* (1.2.23) [Computer software]. https://CRAN.R-project.org/package=jmv

Wickham, H. (2016). *ggplot2: Elegant Graphics for Data Analysis*. Springer-Verlag.

Wickham, H., Averick, M., Bryan, J., Chang, W., McGowan, L., François, R., Grolemund, G., Hayes, A., Henry, L., Hester, J., Kuhn, M., Pedersen, T., Miller, E., Bache, S., Müller, K., Ooms, J., Robinson, D., Seidel, D., Spinu, V., … Yutani, H. (2019). Welcome to the Tidyverse. *Journal of Open Source Software*, *4*(43), 1686. https://doi.org/10.21105/joss.01686
